# cAMP export by the fission yeast *Schizosaccharomyces pombe*

**DOI:** 10.17912/micropub.biology.000384

**Published:** 2021-04-02

**Authors:** Jeremy Eberhard, Charles S. Hoffman

**Affiliations:** 1 Boston College

## Abstract

The fission yeast *Schizosaccharomyces pombe* produces a cAMP signal in response to glucose detection. Previous characterization of this signaling focused on intracellular levels of cAMP. Here, we find that the cAMP is secreted into the medium almost immediately. This is not due to PKA activation as might have been expected. In addition, a strain that is highly deficient in drug efflux shows only a modest reduction in the secretion of cAMP to the growth medium. These observations reveal a previously unappreciated aspect of cAMP metabolism in an important model organism, leading to new questions regarding the mechanism and benefit of cAMP export in *S. pombe*.

**Figure 1. Examination of intracellular and extracellular cAMP levels as a function of culture conditions and genotype:**
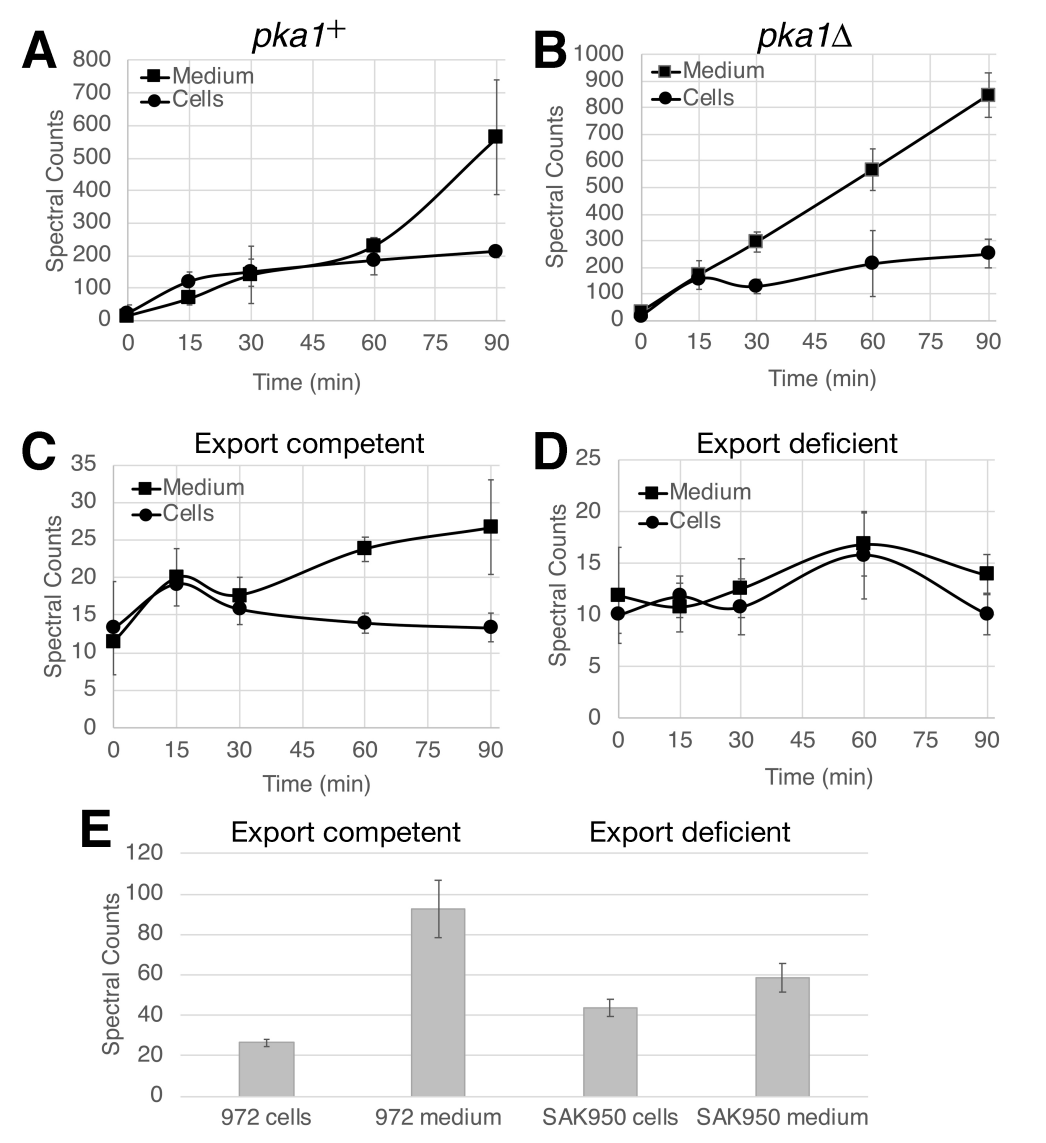
**A)** cAMP response to PDE4 inhibition in *pka1*^+^ strain CHP2027 expressing human AC9, GNAS^R201C^, and PDE4D2. **B)** cAMP response to PDE4 inhibition in *pka1Δ* strain CHP2455 expressing human AC9, GNAS^R201C^, and PDE4D2. **C)** cAMP response to glucose exposure in wild type strain 972. **D)** cAMP response to glucose exposure in strain SAK950 containing seven deletions to reduce drug efflux. **E)** Intracellular and extracellular levels of cAMP in saturated cultures of wild type strain 972 and export deficient strain SAK950 grown in EMM (3% glucose). Values represent the means and standard deviations from three independent experiments in panels A-D, and from four samples from each strain in panel E.

## Description

Signaling by the second messenger cyclic 3’-5’ adenosine monophosphate (cAMP) plays critical roles in most, if not all, organisms. Most of the genes of the *Schizosaccharomyces pombe* glucose-cAMP signaling pathway were identified by mutations that allow transcription of an *fbp1-ura4* reporter that is normally repressed by the cAMP-dependent protein kinase PKA in cells growing under high glucose conditions (Hoffman and Winston 1990). This screening identified eight genes of the pathway, including the *git2*/*cyr1* adenylyl cyclase (AC) gene (Hoffman and Winston 1991). The dynamic change in intracellular cAMP levels in response to glucose exposure was subsequently examined in wild type and mutant strains (Byrne and Hoffman 1993) following a procedure used to study cAMP signaling in budding yeast (Fedor-Chaiken *et al.* 1990). Wild type cells display a rapid response to glucose with a peak at around 20 minutes after glucose addition, followed by a gradual reduction in cAMP levels, while strains carrying mutations in *git1*, *git3*, *git5*, *git7*, *git8* (*gpa2*) and *git10* (*hsp90*) fail to elevate cAMP levels in response to glucose. These genes comprise a G protein-mediated signaling pathway, in which the Gpa2 Gα stimulates AC activity (de Medeiros *et al.* 2013). More recently, we developed platforms for small molecule screening to identify chemical inhibitors of heterologously-expressed cyclic nucleotide phosphodiesterases (PDEs) or ACs, along with mass spectrometry assays to demonstrate a direct impact on cAMP levels (Ivey *et al.* 2008; Getz *et al.* 2019). Once again, we focused on intracellular cAMP levels by collecting cells on a glass filter and releasing cAMP by immersing the filter in 1M formic acid. However, while studying candidate AC inhibitors, we investigated the possibility that a compound could lower intracellular cAMP by causing cAMP to be exported from the cell rather than by inhibiting its synthesis. It was through these experiments, we observed that most of the cAMP was already being exported into the medium. This should not have been a surprise as it had been shown previously that cAMP is present in both *S. pombe* cells and the growth medium in steady state cultures (Schlanderer and Dellweg 1974). Here we examine the dynamics of cAMP export and test whether PKA or a collection of ABC transporters play a role in this process.

To begin, we compared cAMP release from *S. pombe* CHP1852 cells (AC5-expressing cells treated with PDE4 inhibitor Rolipram for 2.5 hours; (Getz *et al.* 2019)) by exposure to 1M formic acid versus a 50% acetonitrile solution (four samples of each from a single culture). This was carried out to determine if our protocol for measuring cAMP levels in a cell culture primarily detects cAMP in the medium, or cAMP in both the cells and the medium. If this protocol releases cAMP from cells, we could avoid the need to evaporate off the formic acid, as required in our previous method. While we observe slightly higher cAMP levels when cell pellets are treated with formic acid (847±120 spectral counts; mean± SD) versus 50% acetonitrile (620±165 spectral counts), at a *p* value of 0.067 in a two-tailed Student’s *t-*test, this difference is not statistically significant. However, even if further repetition of this experiment provided statistical significance to this difference, the modest reduction in cAMP release by acetonitrile treatment would not alter the conclusions from the following experiments examining the ratio of intracellular to extracellular cAMP. This allows for a simple protocol to study the dynamic change in cAMP levels in cells versus the growth medium under two conditions: 1) exposure of strains expressing the human AC9, GNAS^R201C^ and PDE4D2 to the PDE4 inhibitor Rolipram, and 2) exposure of strains with an intact *S. pombe* cAMP pathway to glucose. In addition, we examined whether mutations affecting PKA or a group of ABC transporters affect cAMP export.

Using a pair of strains for which the *S. pombe* AC and PDE have been replaced with the human transmembrane AC9 enzyme, a mutationally activated form of the human Gα_S_ for transmembrane ACs (GNAS^R201C^), and the human PDE4D2 enzyme, we measured intracellular and extracellular cAMP accumulation upon exposure to the PDE4 inhibitor Rolipram (Getz *et al.* 2019). This heterologous system was first examined as these strains produce significantly more cAMP than is found in wild type *S. pombe* strains, thus providing a larger signal from which to make a comparison. Strain CHP2027 has an intact *pka1*^+^ gene, encoding the catalytic subunit of PKA (*S. pombe* possesses a single PKA catalytic subunit gene), while strain CHP2455 carries a deletion of the *pka1* gene. As seen in Figure 1A, there is a rapid elevation in cAMP levels in both cells and the growth medium in the first 15 minutes followed by a gradual elevation for the next 45 minutes. However, from 60 minutes to 90 minutes after Rolipram treatment, the amount of cAMP in the growth medium rises significantly faster than the intracellular cAMP. This might suggest a PKA-triggered export mechanism to reduce intracellular cAMP levels, which would be supported by a reduction in cAMP export in a strain lacking PKA. However, the response to Rolipram in strain CHP2455, lacking PKA activity, does not support this model (Figure 1B). The change in intracellular cAMP levels almost perfectly matches that seen in Figure 1A, however the secreted cAMP levels increase in a more linear fashion. If anything, it appears that PKA activity may cause a retention of intracellular cAMP levels during the first 60 minutes, which seems counterintuitive. Ultimately, by 90 minutes, there is three to four times as much cAMP in the growth medium as in the cells, although due to the relative volume of cells to medium in the culture the intracellular concentration of cAMP is much higher than the concentration of cAMP in the medium.

We next compared cAMP export in a wild type *S. pombe* strain, 972, and strain SAK950 (Aoi *et al.* 2014), which carries seven gene deletions that dramatically reduce drug efflux. These genes encode ABC transporters or proteins that regulate expression of ABC transporters. The ABC transporter superfamily can be divided into seven subfamilies A-G. Members of the C and G subfamilies export cyclic nucleotides in mammalian cells (Cheepala *et al.* 2013), while a member of the B subfamily exports cAMP in the slime mold *Dictyostelium discoideum* (Miranda *et al.* 2015). Notably, of the genes deleted in SAK950 cells, *pmd1* closely resembles a member of the ABC B subfamily, while *bfr1* closely resembles a member of the ABC G subfamily. Initially, we examined the cAMP response to glucose detection. Wild type strain 972 displays a similar response with regard to intracellular levels that we observed previously (Byrne and Hoffman 1993), with a peak at 15 minutes followed by a gradual reduction (Figure 1C), while the amount of cAMP exported to the medium continues to increase. Unfortunately, strain SAK950 grows very poorly in low glucose conditions and shows little to no response to glucose (Figure 1D), thus preventing an assessment of the impact of these deletions on cAMP export in this context. To examine whether these deletions reduce cAMP export to the medium, we instead measured intracellular and extracellular cAMP levels in saturated cultures of strains 972 and SAK950. As seen in Figure 1E, SAK950 cells contain significantly more intracellular cAMP than do 972 cells (*p* < 0.01), while there is significantly less cAMP in the medium of the SAK950 cultures as compared to the 972 cultures (*p* < 0.01). However, these results may not demonstrate a direct role in cAMP export by any of the affected transporters as a substantial amount of cAMP is still exported by SAK950 cells. It should be noted that *S. pombe* possesses other ABC transporter genes, including *abc2*, *abc3*, and *abc4* that resemble ABC C subfamily genes, which may be responsible for cAMP export by SAK950 cells.

We show here that cAMP export occurs within 15 minutes of treatments that elevate intracellular levels, and that while PKA may play a role, it is not to trigger the export of cAMP from the cells. The mechanism of cAMP export remains largely unknown, but may involve multiple ABC transporters. In addition, it remains to be determined whether cAMP export provides some benefit to the cell. Possible roles could be to limit intracellular cAMP levels or to aid in communication between *S. pombe* cells or between *S. pombe* and other organisms growing in a community as would be the case in the wild.

## Methods

**cAMP assays:** Strains were grown to exponential phase in EMM liquid medium in 3ml cultures. For PDE4 inhibition studies, cells were grown in EMM (3% glucose) and treated with the PDE4 inhibitor Rolipram to a final concentration of 40µM. For glucose detection, cells were grown in EMM (0.1% glucose+3% glycerol) and treated with glucose to a final concentration of 100mM. At the indicated times relative to treatment, 0.2ml of the culture was collected and subjected to 30 sec microcentrifugation to separate the cells from the growth medium. The growth medium was transferred to a tube containing 0.2ml acetonitrile. The cell pellets were resuspended in 0.2ml EMM medium after which 0.2ml acetonitrile was added. After 15 minutes, the samples were subjected to a 1 min microcentrifugation at 14,000xg to remove particulate matter from the samples. 75µl of supernatant was removed to tubes containing 75µl mass spec quality water. This was then filtered using a Millipore Multiscreen filter plate, and the filtrate was transferred to a sample vial. cAMP levels were measured on an Agilent 6460 Triple Quad Mass Spectrometer as previously described (Beste *et al.* 2012).

## Reagents

Yeast strains used in this study are described in the table below. Cells were grown with aeration at 30ºC.

Rolipram was purchased from A.G. Scientific and dissolved in DMSO to a working concentration of 40mM. EMM medium lacking glucose was purchased from US Biological and supplemented with either 0.1% glucose+3% glycerol for low glucose conditions or with 3% glucose for standard growth conditions.

**Table:** 

Strain	Genotype	Source
972	*h*^–^	(Leupold 1970)
SAK950	*h*^+^ *leu1* *ura4-D18 ade6-M216 caf5::bsd^R^ pap1-del pmd1-del mfs1-del bfr1-del dnf2-del erg5::ura4^+^*	(Aoi *et al.* 2014)
CHP1852	*h^+^ fbp1::GFP ura4::fbp1-lacZ leu1-32 pap1Δ::ura4^–^ cgs2::PDE4D2 lys2-97::*pJV1*tif-AC5 lys2^+ ^ars1::*pNMT1*-GNAS1^R201C^ LEU2 git2-2::his7^+^*	(Getz *et al.* 2019)
CHP2027	*h^+^ fbp1::GFP ura4::fbp1-lacZ leu1-32 pap1Δ::ura4^–^ cgs2::PDE4D2 lys2-97::pJV1tif-AC9 lys2^+^ ars1::*pNMT1*-GNAS1^R201C^ LEU2^+^ git2-2::his7^+^*	(Getz *et al.* 2019)
CHP2455	*h^–^ fbp1::GFP ura4::fbp1-lacZ leu1-32 pap1Δ::ura4- cgs2::PDE4D2 lys2-97::pJV1tif-AC9 lys2^+^ ars1::*pNMT1*-GNAS1^R201C^ LEU2^+ ^git2-2::his7^+ ^pka1Δ::ura4^+^*	This study
